# Vaginal Microbiota Diversity in Response to Lipopolysaccharide in Gilts Housed Under Three Housing Systems

**DOI:** 10.3389/fgene.2022.836962

**Published:** 2022-04-08

**Authors:** Luana Alves, Francisco José de Novais, Arthur Nery da Silva, Michelle Silva Araujo, Thiago Bernardino, Germana Vizzotto Osowski, Ricardo Zanella, Matthew Lee Settles, Mark A. Holmes, Heidge Fukumasu, Vera Letticie de Azevedo Ruiz, Adroaldo José Zanella

**Affiliations:** ^1^ Department of Veterinary Medicine and Animal Health, Faculty of Veterinary Medicine and Animal Science, University of São Paulo, Pirassununga, Brazil; ^2^ Department of Veterinary Medicine, Faculty of Animal Science and Food Engineering, University of São Paulo, Pirassununga, Brazil; ^3^ Graduation Program in One Health, University of Santo Amaro, São Paulo, Brazil; ^4^ Faculty of Agronomy and Veterinary Medicine, University of Passo Fundo, Passo Fundo, Brazil; ^5^ Director of Bioinformatics Core, UC Davis Genome Center, Davis, CA, United States; ^6^ Department of Veterinary Medicine, School of Veterinary Medicine, University of Cambridge, Cambridge, United Kingdom

**Keywords:** crates, housing systems, metagenomics, Lipopolisaccharide, outdoor housing

## Abstract

The United Kingdom and European Union have banned crates for pregnant sows. However, animals are kept in a restrictive environment for up to four weeks after mating, leading to stress and different responses of the animals’ immune system. Here, we used vaginal flushing of gilts to investigate whether housing systems or an experimental inflammatory challenge with lipopolysaccharide (LPS) can modify the gilt vaginal microbiome. Alpha-diversity indices showed differences in the microbiota of gilts housed under different systems (*q* = 0.04). Shannon alpha-diversity richness was higher in gilts group-housed in pens than in gilts housed in crates (*q* = 0.035), but not higher than in other groups. The relative abundance of the operational taxonomic unit (OTU) (*q* < 0.05) revealed specific differences in housing systems before a LPS or saline (SAL control) challenge. We found different abundances in taxa of *Actinobacteria*, *Bacteroidetes*, *Cyanobacteria*, *Firmicutes*, and *Proteobacteria* in gilts housed in the different systems before challenge. After the LPS challenge, significant differences were detected in the relative abundance of OTUs (*q* < 0.05) for the LPS-challenged group compared with SAL animals for each housing system. The phylum *Staphylococcus* showed higher abundance among the LPS-challenged gilts than in SAL-challenged animals. Furthermore, *Enterobacter* was more abundant in the LPS-challenged gilts housed in crates than in SAL-challenged gilts housed in crates. *Streptococcus suis, Conchiformibius, Globicatella* and *Actinobacillus* were more abundant in LPS-challenged gilts in indoor group housing than in SAL gilts in the same housing system. Gilts kept outdoors did not show changes in vaginal microbiota after an LPS challenge. Gilts housed in crates showed clinical signs of urogenital infection, whereas gilts housed outdoors and in indoor group housing did not. The relationship between environment, immune response, and microbiota suggested that animals in a poor environments experience difficulties responding to a challenge and their vaginal microbiota is altered as a consequence, with decreased richness of normal vaginal microbiota, and increased opportunistic bacteria. Welfare indicators measured by gilts’ responses to housing systems however, do not fully explain mechanisms associated with the unique signature in vaginal microbiota encountered in the different housing systems.

## Introduction

Crate systems are still used for housing pregnant sows due to their easy in handling during estrus detection and early assessment of pregnancy. However, crate systems have come under increased scrutiny due to increased indicators of stress and poor welfare in crate-housed sows (Broom et al., 1995; Rhodes et al., 2005). Crates are still allowed in many countries, even after the European legislation that bans close confinement of sows (EUR-lex, 2020), though the United Kingdom and European Union have banned crates for pregnant sows. Even in these countries, animals are still kept in a restrictive environment for up to four weeks after mating, leading to stress, which is a main factor affecting adaptive responses of the immune system (Grün et al., 2014) such as the number of immunoglobulins and inflammatory cytokines following immunization (Grün et al., 2014), which can impact animals performance.

Adverse environments during the gestation period cause stress, leading to cognitive and emotional disorders and poor welfare of sows, including impaired health and their offspring’s productivity (Baxter, 2000; Charil et al., 2010; Glover, 2011; Green et al., 2011; Coulon et al., 2013; Glover, 2014). The activation of the hypothalamic-pituitary-adrenal axis releases glucocorticoids, which have been associated with several health consequences such as changes in response to pain, exacerbated stress responses, injury, and increased susceptibility to infection (Archie and Theis, 2011). Recent studies have demonstrated that these welfare impairments can have life-long consequences for pregnant females (Coulon et al., 2013; Jašarević et al., 2015; Buffington et al., 2016; Kelly et al., 2016) and may be transmitted to the next generation through epigenetic mechanisms (Nery da Silva et al., 2022).

The role of vaginal and gut microbiota has been studied in stress-related processes and in modulating the gut-brain and hypothalamic-pituitary-adrenal axis (Dinan and Cryan, 2012; Foster and McVey Neufeld, 2013; Jašarević et al., 2015; Luna and Foster, 2015; Kelly et al., 2016; Kennedy et al., 2016; Liang et al., 2022). Several factors, including environment, physiology, genetics, and social relations can modulate the microbial community (Archie and Theis, 2011). Therefore, stressful situations may modify microbiota, influencing resistance and response to pathogenic challenges (Archie and Theis, 2011), such as the number of defense cells in the blood (Rhodes et al., 2005). Although there are studies assessing gilt vaginal microbiota, the vaginal tract is the first contact of a newborn with microbes that will colonize their skin, gut and mucosa, that can cause modify welfare, including health (Dinan and Cryan, 2013; Jašarević et al., 2015; Liang et al., 2022). Several issues, including breeding methods, can also alter vaginal microbiota (Luque et al., 2021). Here, vaginal microbiota flushing samples from gilts housed in crates, indoor group housing, and outdoor group housing were studied to investigate whether housing systems or an experimental inflammatory challenge with lipopolysaccharide can modify the vaginal microbiome.

## Materials and Methods

### Ethics Approval

The experiment was undertaken at the pig farm of the University of São Paulo (USP), located in Pirassununga, Brazil, upon approval of the Ethics Committee on the Use of Animals (CEUA) at the School of Veterinary and Animal Science (FMVZ/USP), under the number 3902100816.

### Experimental Design

Thirty-six gilts (244 ± 22 days old) from a commercial lineage were studied over 10 consecutive days (D1 to D10) as a split-plot 3 × 2 design. On day (D3), the gilts were housed in crates (C) (*n* = 9), indoor group-housing (GH) (*n* = 14), or outdoor group-housing (OD) (*n* = 13). At D6, fifteen animals were challenged intravenously with lipopolysaccharide (LPS) (E. coli O111:B4, Sigma Aldrich^®^, 2 μg/kg) for all housing systems (C-LPS, *n* = 4; GH-LPS, *n* = 6; and OD-LPS, *n* = 5), whereas 21 control animals received 1 ml intravenously of sterile saline (SAL) (C-SAL, *n* = 5; GH-SAL, *n* = 8; and OD-SAL, *n* = 8). In order to assess changes on vaginal microbiome, vaginal flushing was performed the day before the challenge (D5), and 4 days after the challenge (D10). After the completion of the experiment, all animals were euthanized. Five out of the initially fourteen animals kept in crates were removed from the study as a result of the clinical diagnosis of urogenital infection.

All animals were housed in outdoor group-housing within an approximately 2,300 m^2^ paddock before treatment allocation, receiving a commercial diet, 1 kg at 7 a.m. and 1 kg at 4 p.m. The diet was composed of 70% corn, 28% soybean meal, and 2% vitamin-mineral premix. The animals were fed in individual feeding stalls. Water was offered *ad libitum*. Estrous cycles were synchronized in all the animals using 20 mg of altrenogest (Regumate^®^, MSD Saúde Animal, SP, Brazil), in the cycle prior to treatment allocation. Treatment allocation preceded 4 days of expected ovulation.

During the trial period, the 13 OD animals had approximately 2,300 m^2^ to explore. The 14 GH animals were kept in a pen 6.7 m × 4.4 m (3.3 m^2^/gilt) with nine individual feeding stalls (1.8 m × 0.55 m) and a nipple drinker in each stall for *ad libitum* access to water. The 9 C animals were housed in 1.8 m × 0.55 m crates with a nipple drinker and *ad libitum* access to water.

Rectal temperature of all animals was measured at the following eight intervals on LPS/saline challenge on the day immediately before the challenge (T0), and hourly up to seven hours after the challenge (T1- T7).

### Vaginal Microbiota Collection

The vaginal flushing protocol was adapted from Patras and Doran (2016). Vaginal flushing samples were collected at D5 and D10 for each animal, in order to assess the difference in microbial composition before and after the challenge. Prior to sample collection, the vulva was cleaned using neutral soap and water and was dried with a clean paper towel. Vaginal flushing samples were then collected using 20 ml sterile saline solution (0.9%), which was introduced into the animal’s vagina lumen using a sterile urethral catheter attached to a 20 ml sterile syringe. The recovered flushing was stored in 15 ml DNAse and RNAse-free conic tubes (CRAL). The sample was homogenized and divided into two aliquots (1.5 ml), immediately placed in liquid nitrogen and stored at −80°C.

### Vaginal Microbiome Data

DNA from 72 vaginal flushing samples was extracted to profile the vaginal microbiota by 16S rRNA gene sequencing analysis. Bacterial DNA was extracted using the ZymoBIOMICS™ DNA Miniprep Kit^®^ (Zymo Research), following the manufacturer’s instructions. DNA quality was evaluated on a DeNovix^®^ spectrophotometer for quantity (ng/µL) and quality (optical density 260/280 ratio and 260/230 ratio), then stored at −20°C. DNA sequence data were generated using Illumina MiSeq paired-end sequencing Platform with reads of 2 × 250 bp. The library preparation was performed according to Illumina recommendations involving two PCRs, two purification steps, two agarose gels, quantification, normalization, multiplexing, and library denaturation. The first PCR was performed for locus-specific amplification, where primers flanking the V3-V4 region of 444 pb between 341 and 785 pb and overhang adapters (forward overhang, 5′-TCG​TCG​GCA​GCG​TCA​GAT​GTG​TAT​AAG​AGA​CAG-3′; reverse overhang, 5′-GTC​TCG​TGG​GCT​CGG​AGA​TGT​GTA​TAA​GAG​ACA​G-3′) were used. AMPure XP beads were used for purification, and the generated fragments were assessed by agarose gel electrophoresis. The second PCR was used to bond the 96 barcodes of the Nextera XT kit, followed by additional purification and validation steps. A heterogenic control, PhiX fago, was combined with the amplicon pool. Finally, PhiX and library denaturation was performed to allow sequencing.

Sequenced data from thirty-six samples of vaginal flushing on the day before challenge (D5) and after challenge (D10) were assessed. First, demultiplexing sequence reads were pre-processed using dbcAmplicons version 0.9.0. to remove primers, adapters, and low-quality reads. Next, the unmerged forward and reverse reads were imported into QIIME2 version 2019.10, and amplicon sequence variants (ASVs) were determined following the DADA2 analysis pipeline. After the cleanup procedure, 8,703,624 reads were mapped, and 8,919 ASVs were identified. After quality filtering, the sequences were clustered into operational taxonomic units (OTUs). The clustered sequences of prevalence and total abundance were compared using the Silva rRNA reference database (Quast et al., 2012). For analysis purposes, the relative abundance of the ASVs was calculated by dividing the counts of each taxa by the total number of counts for a given sample. Alpha-diversity was obtained, and measured as observed ASVs, Chao1, inverse Simpson, Simpson, Shannon. Beta-diversity was performed using Principal Coordinate Analysis (PCoA) on distance matrix with Bray-Curtis dissimilarity on the weighted Unifrac distance. Analyses of microbiota diversity were performed using Phyloseq Bioconductor package (McMurdie and Holmes, 2013), Vegan R package, Phangorn R package (Schliep et al., 2017) and Decipher Bioconductor package (Wright, 2016).

### Statistical Analysis

In order to assess how the housing systems and challenges affected the difference in microbial composition and diversity, the following linear model was used:

$$y_{ij}=\mu +{\text{HousingSystem}}_{i}+Challenge_{j}+{\left(\text{HousingSystem}*Challenge\right)}_{ij}+\log\left(L_{ijk}\right)+\varepsilon_{ij}$$ (1)

where yijk is the raw count for the ASV analyzed; µ is the overall mean; HousingSystemi is the fixed effect of the “ith” housing system (C, GH and OD); Challengej is the fixed effect of the “jth” challenge (D5 or D10); HousingSystem*Challengeij is the interaction effect of “ith” housing system and “jth” challenge; and log (Lijk) is the trimmed mean of M values (TMM) normalized by library size, used as an offset. The TMM normalization (Robinson and Oshlack, 2010) factors used to normalize library size were obtained based on all the ASVs in the dataset (∼900) and animals. Differential microbiome analyses were considered significant when q-value < 0.05 (Robinson et al., 2010). As a “Bayesian posterior p-value” for multiple testing corrections, “q-value” is an “adjusted p-value” for false-discovery rate (Benjamini, 2010).

Alpha-diversity, a linear mixed model including the fixed effects in Eq. 1 was used, and the offset was removed from the model. A two-way ANOVA was performed for richness and evenness difference, with a Tukey’s honest significant difference test (Tukey’s HSD) post-hoc. Analyses were performed using the stats R package.

Rectal temperature after the LPS challenge was analyzed using a restricted maximum likelihood model, including time and system as fixed effects and animal as random effect, in GraphPad Prism version 9.0.0.

## Results

### The LPS Challenge Increased the Temperature of Gilts

**FIGURE 1:**
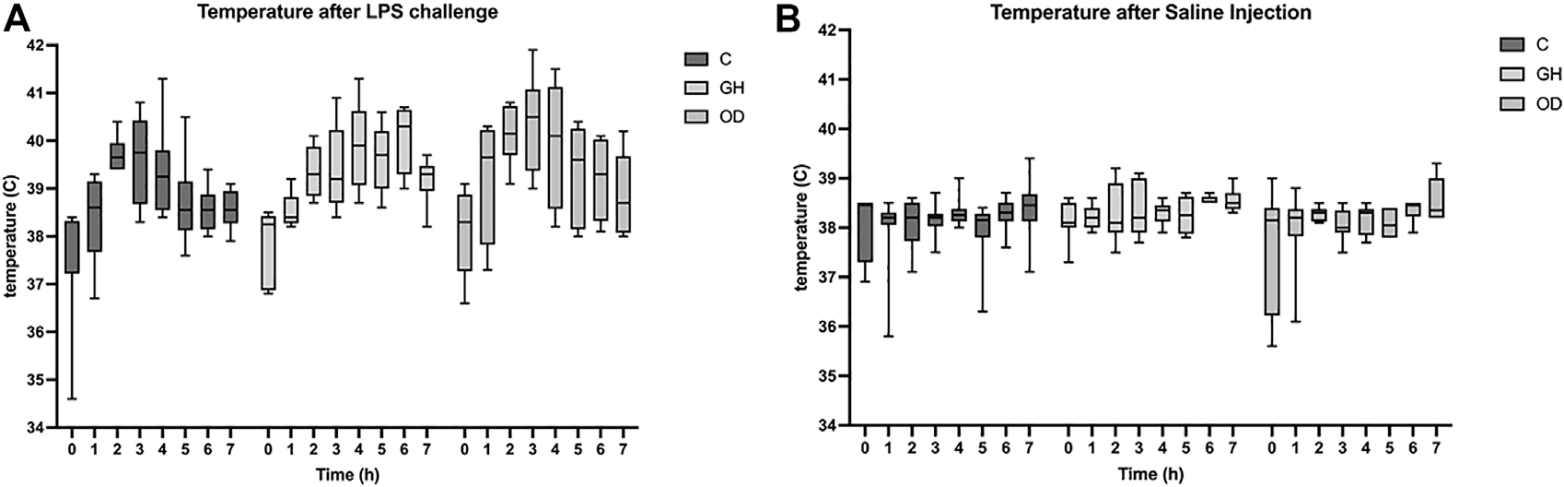
Rectal temperature of gilts measured the day before lipopolysaccharide (LPS) or saline (SAL) challenges (T0) or one hour apart up to 7 h (T1–T7) after LPS challenge **(A)** or SAL challenge **(B)** in indoor group-housing (GH) (*n* = 14), outdoor group-housing (OD) (*n* = 13), and crates (C) (*n* = 9). (*) Statistical significance (*q* < 0.001) in rectal temperature in all groups at 3 h after LPS-challenge, but no housing effect. (**) Statistical significance (*q* < 0.008) in rectal temperature in all groups at 6 h after SAL-challenge, but no housing effect.

After the LPS challenge, a significant increase in rectal temperature was observed in all groups with the higher temperature observed at 3 h (*q* < 0.0001, Figure 1A), but no housing effect was noted. Meanwhile, animals subjected to SAL injection showed an increase in rectal temperature at 6 h (*q* = 0.0069, Figure 1B) with no differences among groups.

### Effects of Housing on Vaginal Microbiome

**FIGURE 2:**
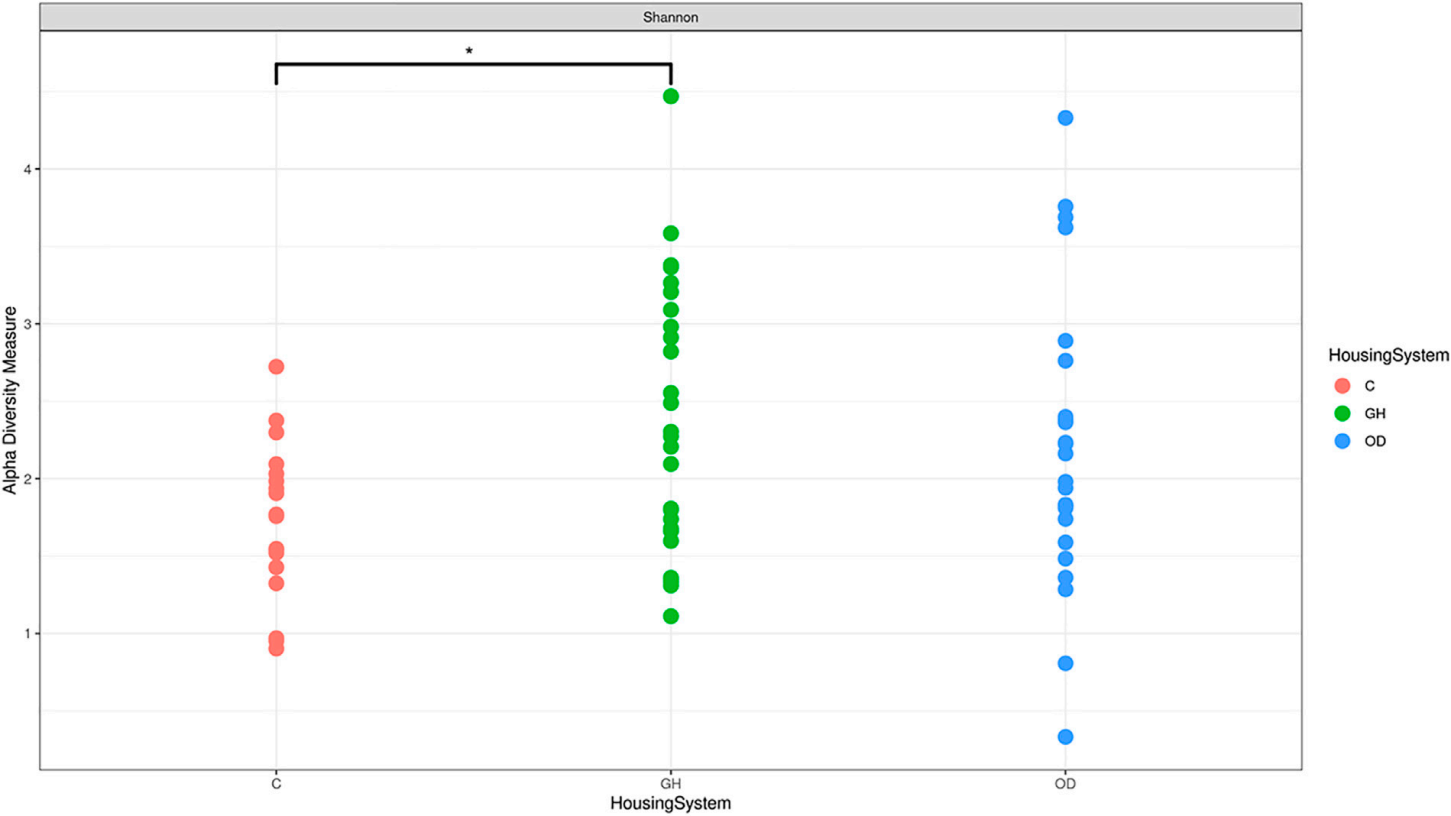
Differences in microbiota Shannon alpha-diversity in crates (C) (*n* = 9), indoor group-housing (GH) (*n* = 14), and outdoor group-housing (OD) (*n* = 13). (*) Statistical significance (*q* = 0.0359) in alpha diversity richness in GH group versus C group.

The gilts housed in different systems showed differences in the vaginal microbiota alpha-diversity (*q* = 0.04), which are illustrated in Figure 2. Differences in richness were detected on group housing (GH) and crates (C) (*q* = 0.035) for Shannon alpha-diversity. There was a higher Shannon richness measurement for GH when compared to C (*q* = 0.0359). However, no differences were observed on beta-diversity, the exploratory ordination plot with log-transformed counts; and Bray-Curtis dissimilarity illustrate beta-diversity among the three housing system groups, or LPS challenge (Supplementary Figure S1).

**FIGURE 3:**
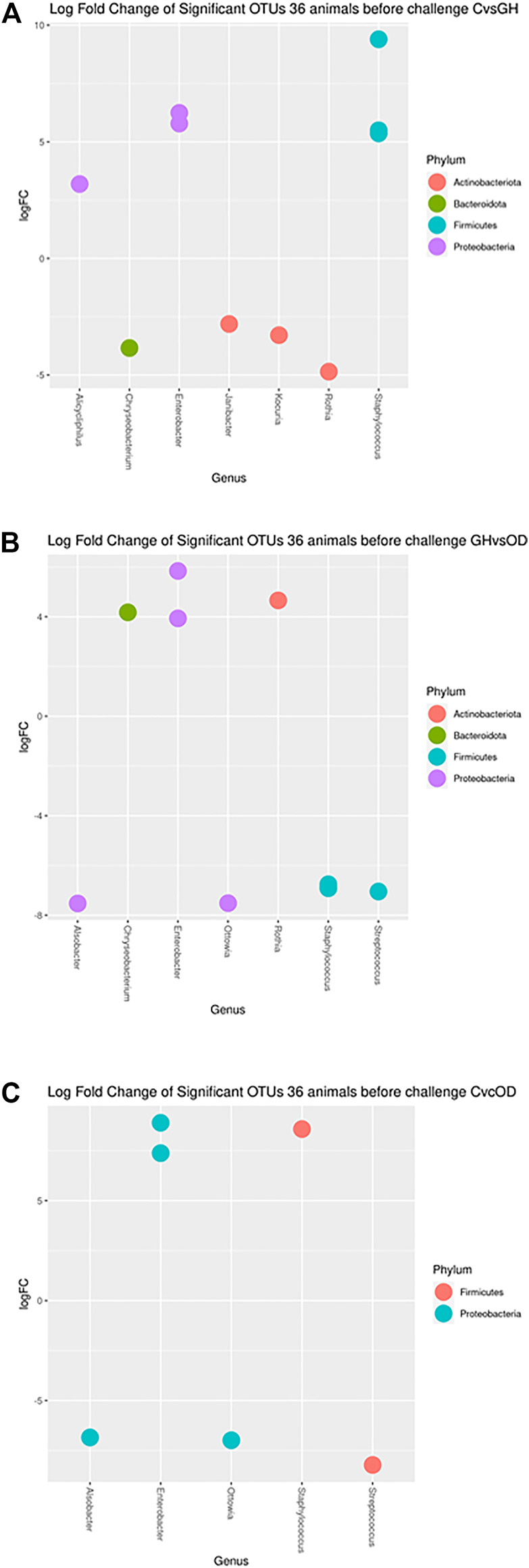
Log fold changes of significant OTUs before LPS/SAL challenge in the different housing systems: crates (C)–*n* = 9; indoor group-housing (GH)–*n* = 14; and outdoor group-housing (OD)–*n* = 13. **(A)** Positive values correspond to C animals, and negative values correspond to GH animals. **(B)** Positive values correspond to GH animals, and negative values correspond to OD animals. **(C)** Positive values correspond to C animals, and negative values correspond to OD animals.

Specific differences in OTUs relative abundances (*q* < 0.05) were found for housing system before the challenge (Figure 3). Contrasts for the housing gilts before the challenge revealed different taxon from phylum *Actinobacteriota*, *Bacteroidota*, *Cyanobacteria*, *Firmicutes*, and *Proteobacteria*. The differential abundance analysis showed the presence of *Staphylococcus sp.* and *Enterobacter sp.* changing in animals of all housing systems.

The taxa of *Staphylococcus sp.*, *Enterobacter sp.,* and *Alicycliphilus sp.* had higher abundance in C compared with GH (*q* < 0.0373; <0.0064; = 0.0373, respectively) (Figure 3A). In contrast, *Janibacter sp., Kocuria sp., Chryseobacterium taklimakanense, Rothia sp.*, and family taxon of *Weeksellaceae* and *Muribaculaceae* were more abundant in the GH group (*q =* 0.0478; = 0.0373; = 0.0164; = 0.0373; = 0.0204; = 0.0186, respectively). The *Enterobacter sp., Rothia sp.,* and *Chryseobacterium taklimakanense* had higher abundance in GH compared with OD (Figure 3B) (*q* < 0.0429; = 0.0125; = 0.001, respectively). The *Staphylococcus sp., Streptococcus sp., Alsobacter sp., Ottowia sp.*, and order taxon of *Chloroplast* were more abundant in animals housed OD (*q* = 0.0059; = 0.0059; <0.0001; <0.0001; <0.0059, respectively). The extreme housing system comparison had *Staphylococcus sp., and Enterobacter sp.* genera as more abundant in C than in OD (Figure 3C) (*q <* 0.0001; <0.0011, respectively). The opposite comparison had *Streptococcus sp., Ottowia sp., Alsobacter sp.*, order taxon of *Chloroplast*, and family taxon *Muribaculaceae* (*q =* 0.0184; = 0.0016; = 0.0016; <0.0398; = 0.0066, respectively) more abundant in OD than in C.

### Vaginal Microbiome Changes After LPS Challenge

**FIGURE 4:**
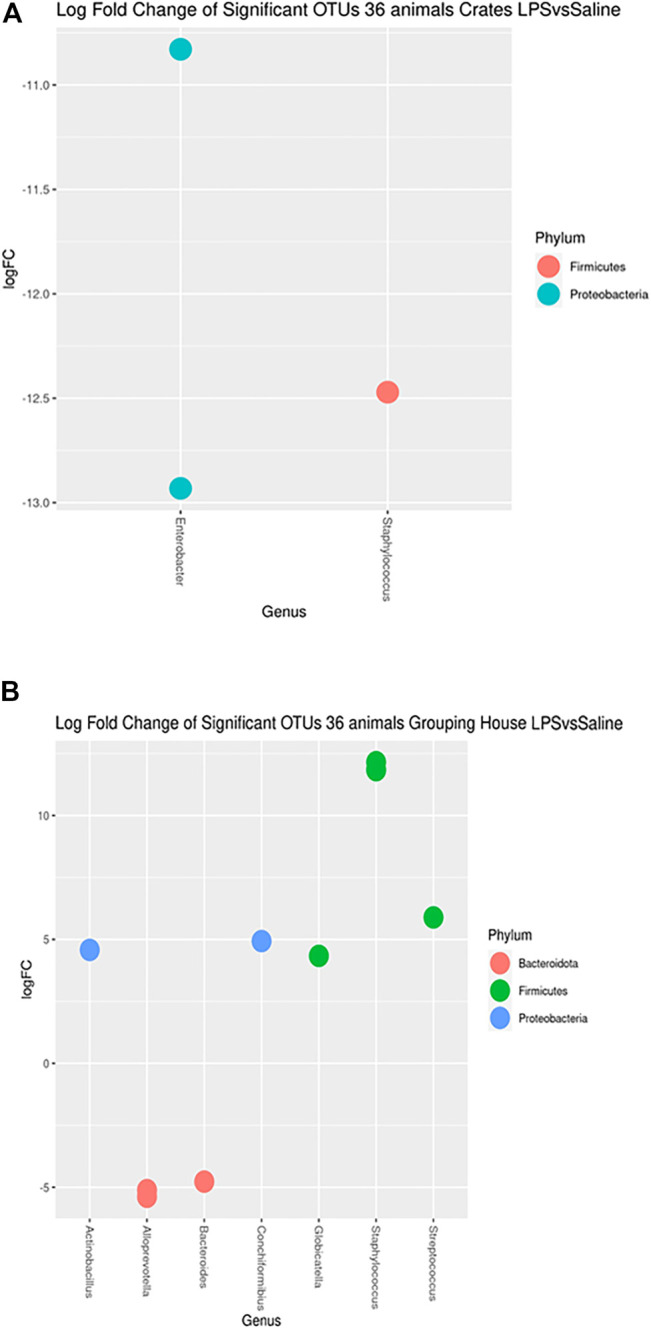
Log fold changes of significant OTUs after LPS or SAL challenge in the different housing systems: crates (C)–*n* = 9; indoor group-housing (GH)–*n* = 14; and outdoor group-housing (OD)–*n* = 13. **(A)** Negative values correspond to C-SAL animals (*n* = 5). **(B)** Positive values correspond to GH-LPS animals (*n* = 6), and negative values correspond to GH-SAL animals (*n* = 8).

After 4 days of the LPS challenge, the vaginal microbiome showed no difference on alpha and beta diversities. In addition, no effect was noted regarding the interaction between the housing systems and LPS challenge on alpha and beta diversities. On the other hand, differences in OTUs relative abundances (*q* < 0.05) were found for the LPS challenge compared to the SAL group for each housing system (Figure 4). Specifically, 3 and 10 OTUs from phylum *Bacteroidota*, *Firmicutes*, and *Proteobacteria* were significantly different for C and GH groups but no phyla were found to be significant different for the OD group after the LPS challenge in comparison to the SAL group.

The taxon of Staphylococcus was more abundant in the crates saline group (Figure 4A) (q = 0.0017). Moreover, Enterobacter were present more in C-SAL than in C-LPS (q = 0.0009). *Streptococcussuis, Conchiformibius sp., Globicatella sp.* and *Actinobacillus sp.* had higher abundance in GH-LPS than in GH-SAL (Figure 4B) (*q =* 0.0210; = 0.0351; = 0.0421; = 0.0462). The opposite comparison had *Staphylococcus, Alloprevotella sp.* and *Bacteroides sp.* more abundant in GH-SAL than in GH-LPS (*q <* 0.0001; <0.0421; = 0.0462, respectively).

## Discussion

Microbiome changes have been associated with several reproductive alterations, stress, and diseases in humans and other animal species (Sanglard et al., 2020). These alterations affect and modify the existent interplay between the microbiome and mammalian immune system (Borgogna and Yeoman, 2017). Here, we demonstrate the impact of different housing systems associated with a challenge with LPS to simulate a disease process, on the vaginal microbiome.

The current study data were only collected in non-pregnant animals but we anticipate that the differences in vaginal microbiota reported among the experimental groups would be likely to be maintained, or even exacerbated, in pregnant animals. Previous studies have demonstrated that vaginal microbiota of humans have a relationship with the development of microbiota in the newborn (Reid et al., 2011). Miller et al. (2016) suggested an abundance of *Lactobacillaceae* in the vaginal canal in humans plays an important role in avoiding microbiota disequilibrium (Miller et al., 2016). However, mammal’s vaginal microbiota is not similar to that of humans in this aspect (Miller et al., 2016). As expected, in our results we did not find different abundance of *Lactobacillaceae.*

Our results corroborate the studies of Lorenzen et al. (2015), who reported the similarity between the fecal microbiota and the vaginal microbiota, as the most prevalent families of bacteria in vaginal microbiota were also found in gut microbiota. Lorenzen et al. (2015) demonstrated that the swine vaginal microbiota shows an abundance of the phyla *Firmicutes*, *Proteobacteria*, *Bacteroidota*, and *Actinobacteria*. We also found *Fusobacteria* in our data. This is a normal commensal bacterium found in mucosal layers such as in the gastrointestinal tract (Booth, 2007), even if sometimes associated with gastric ulceration (De Witte et al., 2018; De Witte et al., 2019). *Cyanobacteria*, Gram-negative bacteria (Sinha and Häder, 2008) that obtain energy *via* photosynthesis, were also encountered. Chloroplasts were more abundant in outdoor housing system before the challenge in all contrasts of comparison. The contact with the grass and soil available for these gilts in the outdoor system may altered their microbiota. Thus, this finding may be caused by an environmental contamination despite all the care to avoid samples contamination during collection.

Pathogenic and opportunistic bacteria, members of the taxa *Streptococcus, Staphylococcus* and *Enterobacter*, were found in all housing systems. These data have important practical relevance, since urogenital infection is commonly observed in crated animals, meaning that other factors made animals kept in crates more susceptible to urogenital infections. In this case, the presence of the pathogen cannot always be associated with the onset of urogenital tract infections. These findings support our hypothesis that the environment in which the animals are housed is important in that it is likely to compromise the animal’s immunity. Amabebe and Anumba (2018) corroborate our findings, highlighting that a long period of stress exposure with activation of hypothalamic-pituitary-adrenal axis may be harmful for female lower genital tract microbiome resulting in high risk of genitourinary infections.

Considering the important role of hyperthermia in combating diseases, Liu et al. (2019) characterized the systemic effect of LPS as an acute inducer of the inflammatory response in pigs of different genetic lines. In our study, all the animals challenged with LPS showed a similar increase in rectal temperature, indicating a successful immune activation and comparable host defense in crated, group-housed, and outdoor-kept sows. Moreover, LPS use as a surrogate of a pathogenic insult did not show any long-term clinical signs of illness in animals.

Our data demonstrated that housing systems affected vaginal microbiota, creating a unique signature of the microbiota. This environmentally induced vaginal microbiome signature needs further studies to assess the consequences for the health of the gilts and of their offspring. Interestingly, crated gilts demonstrated less variability in the vaginal microbiota, suggesting a system more vulnerable to potential disequilibrium.

These results corroborate our initial hypothesis that vaginal microbiota is affected by the gilt’s housing system. Interestingly, the microbiota of the gilts kept outdoors was not affected by the LPS challenge, whereas both gilts kept in crates and kept in indoor group housing did show a significant effect of LPS challenge in the vaginal microbiota. Animals housed outdoors have better welfare than animals kept in crates and in indoor groups, and their good welfare appeared to make them more resilient to the disease simulation using LPS. However, this mechanism involves more than just the housing system of animals, indicating the need for further studies to determine impacts on animal’s welfare and their susceptibility to disease.

## Conclusion

Our results demonstrate that the vaginal microbiota of gilts depends greatly on the bacterial exposure from the housing environment. In addition, an animal’s response to a disease challenge, represented by LPS inoculation, also varied according to the housing system. LPS challenge did not change the vaginal microbiota in gilts kept outdoors. Interestingly, 5gilts housed in crates showed clinical urogenital infections, commonly caused by gram-negative bacteria, whereas animals housed in indoor or outdoor systems did not. Veterinarians immediately evaluated the gilts that manifested clinical symptoms.

Finally, the diversity of gilts’ vaginal microbiota appears to be affected by the environments where they were kept. Future studies are needed to better understand the interaction between the environment, hormones, other welfare impairments and microbiota.

## Data Availability

The datasets presented in this study can be found in online repositories. The names of the repository/repositories and accession number(s) can be found below: https://www.ncbi.nlm.nih.gov/, PRJNA803177.

## Supplementary Material

Supplementary Figure S1 The exploratory ordination plot with log-transformed counts and weighted Unifrac distance, before challenge **(A)**; after challenge **(B)**.
