# Supplementary figures and images for: Vaginal Microbiota Diversity in Response to Lipopolysaccharide in Gilts Housed Under Three Housing Systems
Source: Front Genet. 2022 Apr 8;13:836962. doi: 10.3389/fgene.2022.836962 (PMC9024362; doi:10.3389/fgene.2022.836962)

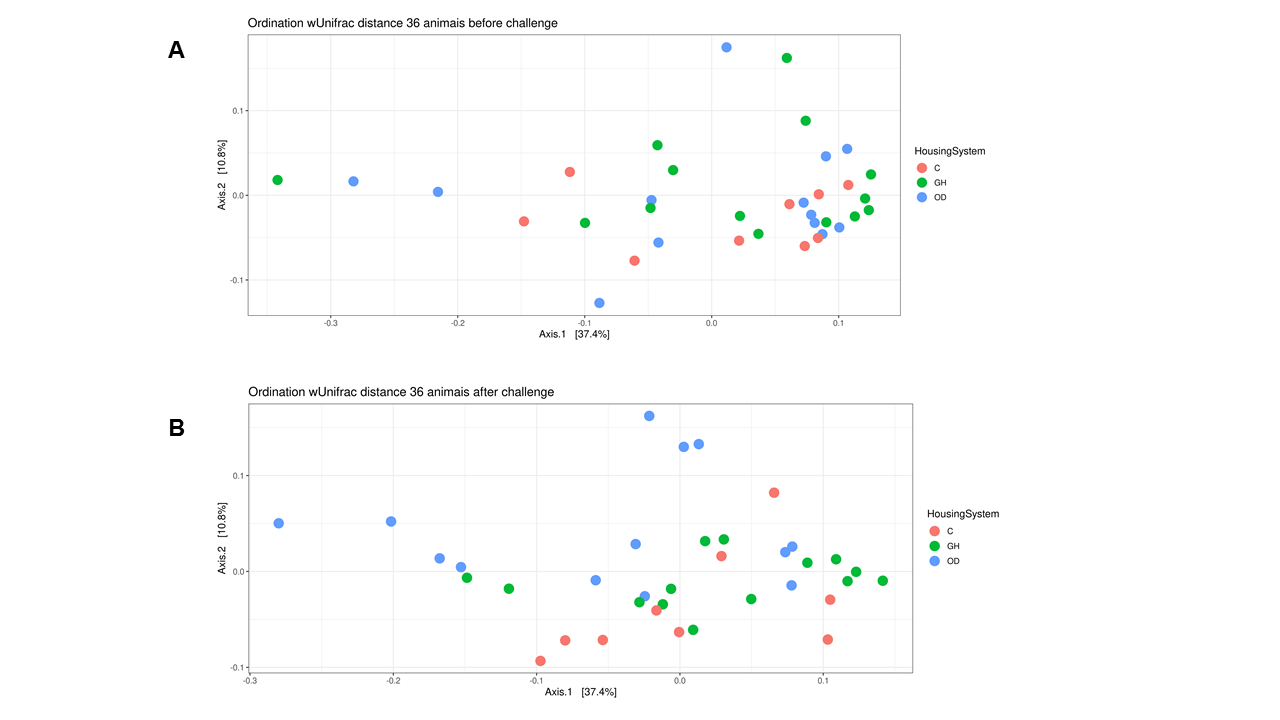

Supplement: Supplementary file 1 [file Image1.TIF]
